# ERAP2 as a potential biomarker for predicting gemcitabine response in patients with pancreatic cancer

**DOI:** 10.18632/aging.204324

**Published:** 2022-10-08

**Authors:** Pian Yu, Shifu Luo, Jiaxin Cai, Jie Li, Cong Peng

**Affiliations:** 1 The Department of Dermatology, Xiangya Hospital, Central South University, Changsha 410000, Hunan, China; 2 National Engineering Research Center of Personalized Diagnostic and Therapeutic Technology, Xiangya Hospital, Changsha 410000, Hunan, China; 3 National Clinical Research Center for Geriatric Disorders, Xiangya Hospital, Changsha 410000, Hunan, China; 4 Hunan Key Laboratory of Skin Cancer and Psoriasis, Xiangya Hospital, Changsha 410000, Hunan, China; 5 Hunan Engineering Research Center of Skin Health and Disease, Xiangya Hospital, Changsha 410000, Hunan, China; 6 Xiangya Clinical Research Center for Cancer Immunotherapy, Central South University, Changsha 410000, Hunan, China; 7 Department of Pharmacy, The Second Xiangya Hospital, Central South University, Changsha 410011, Hunan, China

**Keywords:** pancreatic cancer, drug resistance, gemcitabine, GDSC, ERAP2

## Abstract

Objective: Pancreatic cancer is one of the most malignant tumors, with rapid metastasis, high mortality rate, and difficult early screening. Currently, gemcitabine is a first-line drug for pancreatic cancer patients, but its clinical effect is limited due to drug resistance. It is particularly important to further identify biomarkers associated with gemcitabine resistance to improve the sensitivity of gemcitabine treatment.

Methods: Drug sensitivity data and the corresponding transcript data derived from the Genomics of Drug Sensitivity in Cancer (GDSC) database for correlation analysis was adopted to obtain genes related to gemcitabine sensitivity. Moreover, the survival model of pancreatic cancer patients treated with gemcitabine in The Cancer Genome Atlas (TCGA) database was utilized to obtain key genes. Multiple *in vitro* assays were performed to verify the function of the key biomarker.

Results: Endoplasmic Reticulum Aminopeptidase 2 (ERAP2) was identified as a biomarker promoting gemcitabine resistance, and its high expression resulted in a worse prognosis. Besides, gemcitabine significantly increased the mRNA and protein levels of ERAP2 in pancreatic cancer cells. Additionally, ERAP2 knockdown suppressed tumorigenesis and potentiated gemcitabine-induced growth, migration and invasion inhibition in human pancreatic cancer cells.

Conclusions: ERAP2 may be a novel key biomarker for gemcitabine sensitivity and diagnosis, thus providing an effective therapeutic strategy for pancreatic cancer treatment.

## INTRODUCTION

Pancreatic cancer is a deadly disease with almost the same mortality and morbidity [1, 2]. Pancreatic cancer is difficult to detect in most pancreatic cancer patients because it has no symptoms before it progresses to advanced stages [3, 4]. Almost 90% pancreatic malignancies are pancreatic ductal adenocarcinomas (PDAC) [5]. Patients with advanced pancreatic cancer are routinely treated with radiation and chemotherapy [6]. Despite decades of efforts to improve diagnostic techniques, surgery, radiation therapy and chemotherapy, the overall prognosis for PDAC patients remains poor [7, 8]. Gemcitabine is a deoxycytidine analog widely used for chemotherapy in various solid tumors [9–11], which has been a standard chemotherapy drug for pancreatic cancer patients in the past few decades [12]. Although gemcitabine can prolong the survival period of pancreatic cancer patients, the resistance of pancreatic cancer to gemcitabine hinders its efficacy, which makes pancreatic cancer more difficult to cure [13]. Generally, chemical resistance is divided into intrinsic resistance and acquired resistance [14, 15]. Intrinsic resistance occurs when the treatment is ineffective at the outset, and acquired resistance occurs after several rounds of treatment with anticancer drugs [16]. Nevertheless, neonatal or acquired resistance is the main reason of disease progression and a major obstacle that clinicians have to overcome [17]. Gemcitabine resistance may be mediated through several mechanisms such as the NF-kB pathway, histone deacetylation, heat shock proteins, fatty acid and sphingolipid metabolism, and pyruvate metabolism [13, 18]. However, there is still no effective strategy and target to reverse gemcitabine resistance. Therefore, the mechanism of gemcitabine resistance needs to be further elucidated to benefit more pancreatic cancer patients.

As is known to all, the Genomics of Drug Sensitivity in Cancer database (GDSC database) is the largest public resource for information on drug sensitivity and molecular markers of drug response in cancer cells [19]. The GDSC database collected data on the sensitivity and response of tumor cells to drugs. The variation of cancer genome will greatly affect the clinical therapeutic effect, and the response of different targets to drugs is also diverse. Therefore, GDSC database is very important for the discovery of potential tumor therapeutic targets. Additionally, The Cancer Genome Atlas database (TCGA database) represents a key milestone in the National Cancer Institute’s mission to reduce the burden of cancer suffering, which is not only rich in transcription data, but also has detailed clinical data and is a treasure trove of cancer researchers [20]. TCGA database is conducive to excavating the multi-omics data of various cancers and exploring the molecular mechanism of tumor occurrence and development.

In this study, we employed the drug sensitivity data in GDSC combined with TCGA database to identify Endoplasmic Reticulum Aminopeptidase 2 (ERAP2) as the key gene associated with cellular gemcitabine sensitivity and patient prognosis, providing a new option for adjuvant gemcitabine chemotherapy for pancreatic cancer.

## RESULTS

### Drug sensitivity and transcripts in pancreatic cell lines

Firstly, we downloaded the sensitivity data of pancreatic cancer cell lines treated with gemcitabine from the public GDSC data, which included two data sets: GDSC1 and GDSC2. In this part, GDSC1 data set was adopted to screen genes related to gemcitabine sensitivity, which included IC50 values of 30 pancreatic cancer cells treated with gemcitabine (Table 1). Then, we conducted correlation analysis between gene expression value and IC50 value. We found that the IC50 of gemcitabine was positively relevant to 359 genes and negatively correlated with 456 genes (*p* < 0.05).

**Table 1:** IC50 values of pancreatic cancer cells treated with gemcitabine in GDSC1.

**CELL_LINE_NAME**	**DRUG_NAME**	**Log2_IC50**
AsPC-1	Gemcitabine	1.916122
BxPC-3	Gemcitabine	-5.32043
CAPAN-1	Gemcitabine	-6.23895
CAPAN-2	Gemcitabine	1.773473
CFPAC-1	Gemcitabine	-8.24857
DAN-G	Gemcitabine	3.014603
HPAC	Gemcitabine	-6.48888
HPAF-II	Gemcitabine	-2.22445
Hs-766T	Gemcitabine	1.777713
HuP-T3	Gemcitabine	-3.30747
HuP-T4	Gemcitabine	-5.85751
KP-1N	Gemcitabine	-4.74144
KP-2	Gemcitabine	-2.83739
KP-4	Gemcitabine	-6.09128
MIA-PaCa-2	Gemcitabine	-5.94014
MZ1-PC	Gemcitabine	-1.67868
PANC-02-03	Gemcitabine	-1.38525
PANC-03-27	Gemcitabine	-7.23895
PANC-04-03	Gemcitabine	-1.63365
PANC-08-13	Gemcitabine	-0.71317
PANC-10-05	Gemcitabine	-5.53156
PA-TU-8902	Gemcitabine	-1.79899
PA-TU-8988T	Gemcitabine	-6.14642
PL18	Gemcitabine	-5.27889
PL4	Gemcitabine	-2.83953
PSN1	Gemcitabine	-9.78819
SU8686	Gemcitabine	-4.39629
SUIT-2	Gemcitabine	-7.11558
SW1990	Gemcitabine	-3.4139
YAPC	Gemcitabine	4.533963

### Univariate Cox survival analysis of gemcitabine sensitivity-related genes

We have identified a total of 815 gemcitabine sensitivity-related genes and annotated them with Gencod.v22 gene annotation file, but only 807 were successfully annotated. Then the TCGA-PAAD data set of pancreatic cancer patients was downloaded from the UCSC XENA database, including gene expression sequencing data (FPKM format), survival time, survival status, and gemcitabine medication information. 68 cases of patients with pancreatic cancer treated with gemcitabine were identified through data screening, and their corresponding gene expression sequencing data, survival time and survival status were obtained. After data cleaning and collating, 815 gemcitabine sensitivity relevant genes were selected for univariate Cox survival analysis using the Survival package of R software to determine the influence of gene expression on the patients. In order to establish the multi-factor Cox regression model, we selected the most significant 20 genes for variable screening (Table 2).

**Table 2:** The 20 most significant genes in univariate Cox survival analysis.

**Genes**	**HR**	**z**	**P-value**
STOM	0.407651	-3.09896	0.001942
CACNA2D3	0.118199	-3.03203	0.002429
CD1D	0.447079	-3.03028	0.002443
PSTPIP1	0.325104	-3.02635	0.002475
CDIP1	0.332236	-2.98929	0.002796
DOCK11	0.375723	-2.94056	0.003276
CYB561D1	0.232145	-2.91977	0.003503
RRAGD	0.290525	-2.87705	0.004014
PTK6	1.793435	2.864009	0.004183
SERPINB5	1.84428	2.854813	0.004306
STK3	4.633022	2.833259	0.004608
RP11-89K11.1	0.124358	-2.82909	0.004668
CD99L2	0.312071	-2.8289	0.004671
PCDH1	2.283409	2.825165	0.004726
E2F7	2.602376	2.806041	0.005015
PEX13	3.744544	2.801468	0.005087
ERAP2	1.550369	2.77102	0.005588
UCHL1	0.583004	-2.74929	0.005972
OR2B2	3.77E+14	2.699686	0.00694
PPM1M	0.325905	-2.6741	0.007493

### A multiple factors Cox proportional hazards regression model was established based on Lasso regression analysis

**Figure 1:**
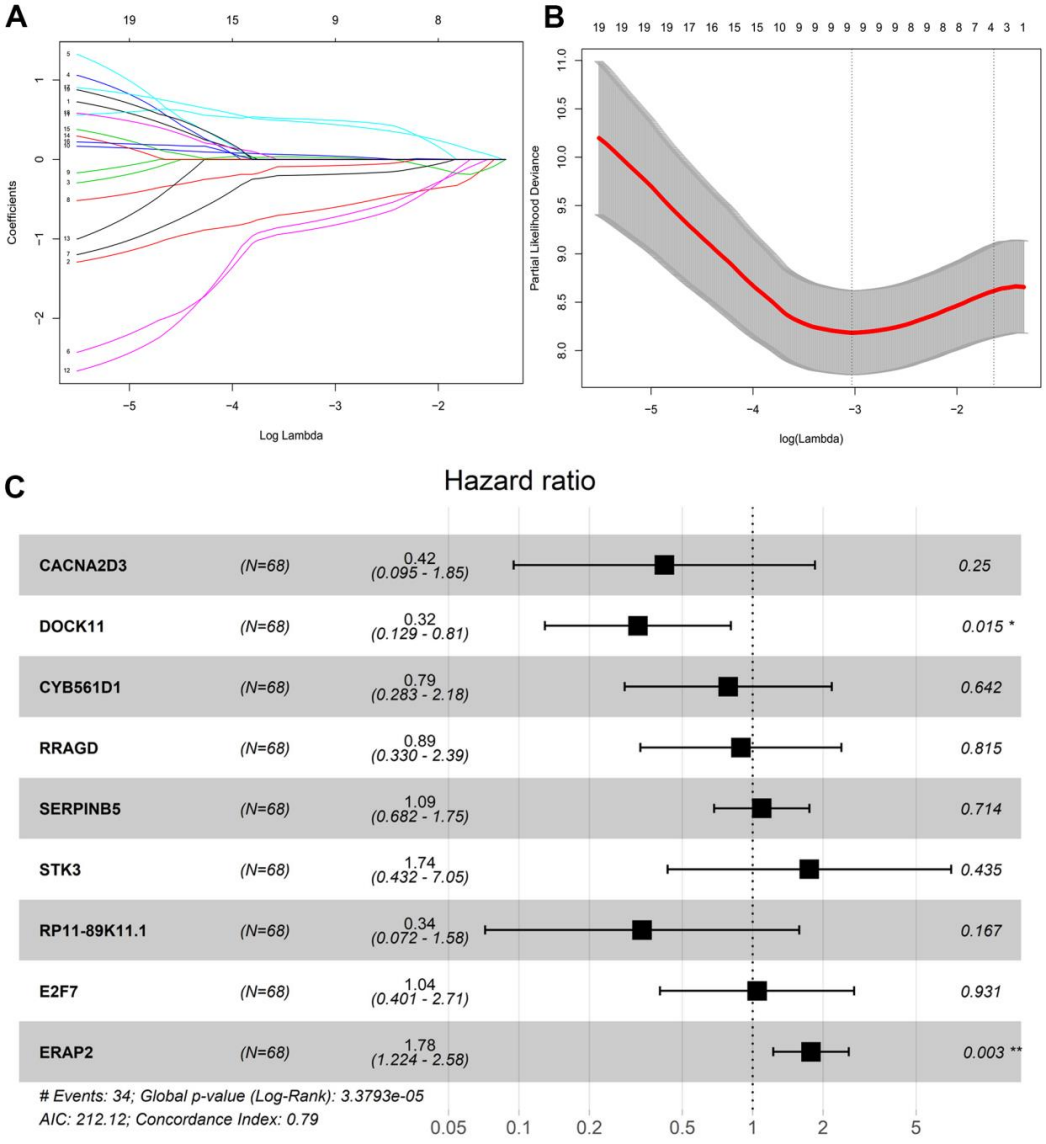
**Lasso regression screening survival model variables.** (**A**) Variable coefficient corresponding to the model with different number of variables. (**B**) 1000 models obtained through ten-fold cross validation. (**C**) Risk coefficient and P value of corresponding genes in multivariate Cox proportional risk regression model.

Next, we excluded OR2B2 for its barely expression, and finally 19 genes entered the variable selection of the model. Additionally, we utilized Lasso algorithm to screen out the independent variables. 1000 models were calculated by setting the parameter nlambda = 1000, and the coefficients of independent variables were obtained (Figure 1A). Besides, we filtered the lambda value (λ value) through 10-fold cross-validation, and obtained two models. It should be mentioned that one model was based on lambda.min (the error mean is the minimum corresponding lambda, dotted line on the left); the other was based on lambda.1se (the maximum lambda of the error mean within 1 standard deviation of the minimum value, dotted line on the right). As shown in Figure 1B, we adopted 9 genes as model variables for model construction. After determining the model variables, we employed the R package survival to construct the multi-factor Cox risk ratio model for these 9 genes, and identified the final model, which included the coefficients, risk values, and P values of each variable. Meanwhile, the coefficients of the best model of Lasso were also shown (Table 3). We adopted R package to plot the Forest plot of this model, and found that the P values of ERAP2 and DOCK11 were both less than 0.05, suggesting that they could be independent prognostic factors for pancreatic cancer patients treated with gemcitabine, respectively (Figure 1C). Moreover, we proved the Concordance Index of this model reaches 0.79, indicating that the model is accurate and reliable.

**Table 3:** Multivariate Cox proportional risk regression model.

**Symbol**	**coef**	**exp(coef)**	**z**	**Pr(>|z|)**
CACNA2D3	-0.87144	0.41835	-1.15058	0.249905
**DOCK11**	-1.13124	0.322634	-2.42187	**0.015441**
CYB561D1	-0.24186	0.785165	-0.46466	0.642175
RRAGD	-0.11797	0.888722	-0.23356	0.81533
SERPINB5	0.087906	1.091886	0.366625	0.713899
STK3	0.556078	1.74382	0.780509	0.435092
RP11-89K11.1	-1.08972	0.33631	-1.381	0.16728
E2F7	0.042367	1.043277	0.086883	0.930765
**ERAP2**	0.573894	1.775166	3.02303	**0.002503**

### Risk factor association analysis

**Figure 2:**
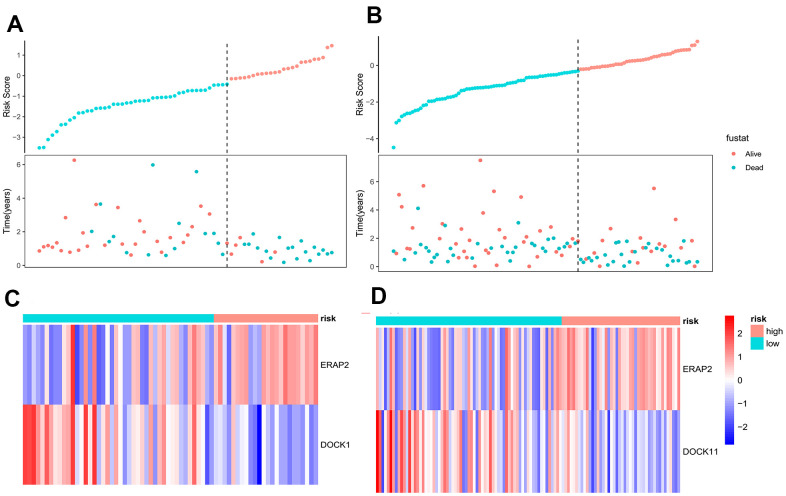
**Correlation analysis of risk factors between model group and validation group.** (**A**) The survival time and survival status of pancreatic cancer patients in the model group as a function of their risk values. (**B**) The survival time and survival status of pancreatic cancer patients in the validation group as a function of their risk values. (**C**) Heat map of gene expression as a function of risk values in the model group. (**D**) Heat map of gene expression as a function of risk values in the validation group.

To further illustrate the validity of the Lasso-Cox regression model, we described the survival time and survival status of patients by a risk factor correlation diagram as their risk values changed. First of all, patients were assigned to high-risk group and low-risk group with cut off value equal to -0.3 (dotted line in the middle), which was calculated by Xtile software. We also mapped the distribution of survival time and status in pancreatic cancer patients based on the distribution of risk values. Patients in the high-risk group of the model group and the validation group showed shorter survival time and lower survival rates compared with the lower risk group (Figure 2A, 2B). We found ERAP2 and DOCK11 were important independent prognostic factors, and the relationship between their expression levels and risk values was also analyzed. As shown in the heat map, ERAP2 expression as a risk factor was higher in the high-risk group, while DOCK11 expression as a protective factor was lower in the low-risk group. The trend of model group and validation group is consistent, indicating a strong universality of the model (Figure 2C, 2D).

### Kaplan-Meier survival analysis based on risk score of pancreatic cancer patients

**Figure 3:**
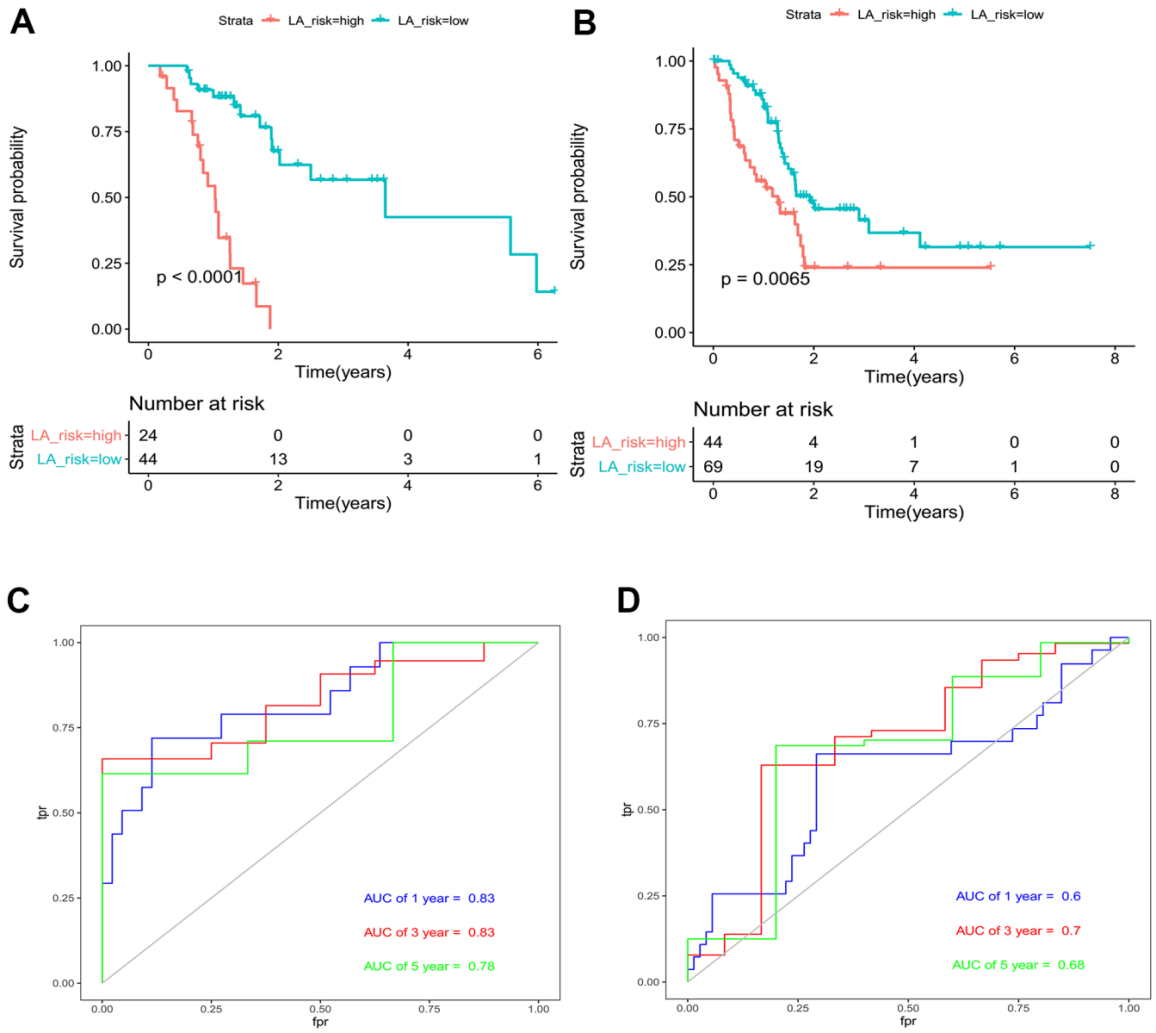
**Evaluation and validation of Lasso-Cox regression model for pancreatic cancer.** (**A**) Risk-based Kaplan-Meier survival curve for 68 pancreatic cancer patients treated with gemcitabine. (**B**) Risk-value Kaplan-Meier survival curve for 113 pancreatic cancer patients with no medication information. (**C**) AUC area of pancreatic cancer patients in the model group at 1, 3 and 5 years. (**D**) AUC area of pancreatic cancer patients in the validation group at 1, 3 and 5 years.

According to the variable coefficients of the optimal model of Lasso regression mentioned above (Table 3), the Lasso regression coefficients corresponding to 9 genes were obtained. Then we obtained the Risk Score of each pancreatic cancer patient according to the expression levels of 9 genes, and obtained the value of risk for 68 pancreatic cancer patients with gemcitabine treatment. After calculating the cut off value by Xtile software, patients with a risk value above -0.3 were considered to be in the high-risk group, while the rest were considered to be in the low-risk group. The corresponding Kaplan-Meier survival curve was shown in Figure 3A. At the same time, 113 pancreatic cancer patients without gemcitabine medication information were applied to validate the model. Similarly, the risk value of each patient was calculated by the expression levels of 9 genes, and the corresponding Kaplan-Meier survival curve was plotted (Figure 3B). It was obvious that the prognosis of low-risk patients in the model group (n = 68) and validation group (n = 113) was better than that of high-risk patients. We further draw the ROC curves of the prediction results of the model group and the validation group, and the 1-, 3- and 5-year ROC curves of pancreatic cancer patients, respectively. In the model group, the 1-, 3- and 5-year AUC area was 0.83, 0.83, and 0.76, respectively, indicating the good predictive ability on the survival of pancreatic cancer patients treated with gemcitabine of the model, and we observed that the Lasso-Cox regression model composed of these 9 genes had a very high accuracy (Figure 3C). In the validation group, the 1-, 3- and 5-year AUC areas were 0.6, 0.7 and 0.68, respectively. In the case of using external data, the model still maintained a high accuracy, indicating that the Lasso-Cox regression model constituted by these 9 genes had good universality (Figure 3D).

### The expression of ERAP2 was associated with gemcitabine sensitivity and response

**Figure 4:**
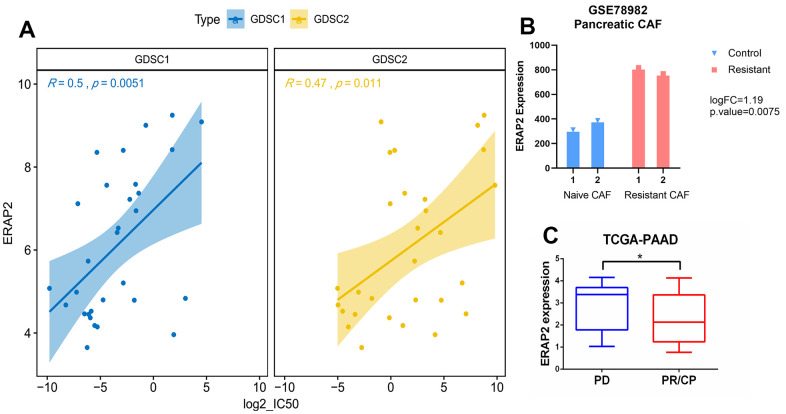
**Relationship between expression of ERAP2 and sensitivity to gemcitabine in pancreatic cancer.** (**A**) Correlation between the expression value of ERAP2 in GDSC1 and GDSC2 pancreatic cancer cell lines and the IC50 value of gemcitabine. (**B**) Expression of ERAP2 in two types of pancreatic cancer fibroblasts. (**C**) Relationship between the effect of gemcitabine treatment and ERAP2 expression in TCGA pancreatic cancer patients.

From the above Cox proportional risk regression model, we identified two genes, ERAP2 and DOCK11, associated with gemcitabine sensitivity. As a risk factor, ERAP2 expression is positively relevant to the risk value of pancreatic cancer patients, and is more suitable as a target for further research. First, we analyzed the relationship between ERAP2 expression and gemcitabine IC50 in pancreatic cancer cells in the GDSC1 and GDSC2 datasets. In GDSC1, the correlation between ERAP2 and gemcitabine log2 (IC50) value reached 0.5. In GDSC2, the correlation between ERAP2 and gemcitabine log2 (IC50) value also reached 0.47 (Figure 4A). In addition, we downloaded the expression chip GSE78982 of pancreatic cancer fibroblast from the GEO database, and analyzed the data by the GEO2R online tool. ERAP2 expression was significantly up-regulated in the gemcitabine resistant group, with differential multiple LogFc value of 1.19 and p-value of 0.0075 (Figure 4B). More importantly, we screened gemcitabine treated patients in the TCA-PAAD pancreatic cancer data set and measured their treatment efficacy, and found significantly lower ERAP2 expression values in the effective group (PR/CR) than in the ineffective group (PD) (Figure 4C). These results suggested that ERAP2 expression was significantly related to gemcitabine sensitivity in pancreatic cancer.

### ERAP2 promoted pancreatic cancer progression

**Figure 5:**
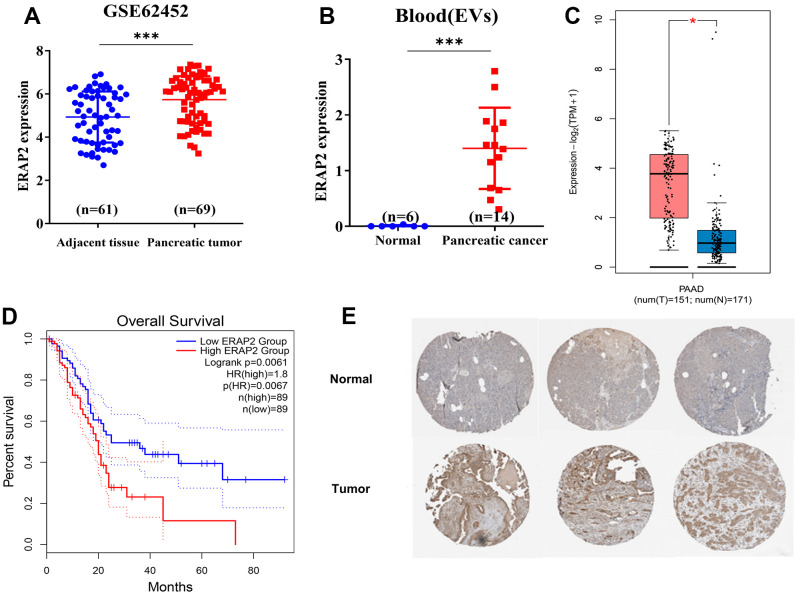
**Expression and prognosis of ERAP2 in pancreatic cancer.** (**A**) Expression of ERAP2 in GSE45452 chip pancreatic tumor and paracancerous tumor. (**B**) The expression of ERAP2 in extracellular vesicles of blood origin. (**C**) Expression of ERAP2 in the pancreatic cancer dataset from GEPIA database. (**D**) Kaplan-Meier survival curve of ERAP2 in pancreatic cancer data set from GEPIA database. (**E**) Immunohistochemical images of ERAP2 in pancreatic tissues and pancreatic tumors from ProteinAtlas database.

The above findings show that ERAP2 is a key gene affecting gemcitabine resistance in patients with pancreatic cancer, which enable us to further study whether there is a relationship between ERAP2 and the diagnosis and prognosis of pancreatic cancer. We downloaded the expression microarray GSE62452 from GEO database of pancreatic tumors and paracancerous tumors, and found that ERAP2 was highly expressed in pancreatic tumors (Figure 5A). In addition, ERAP2 was also significantly overexpressed in blood extracellular vesicle samples of pancreatic cancer patients from the BBCancer database (Figure 5B). Next, we analyzed the ERAP2 expression in pancreatic cancer by the GEPIA database and found that ERAP2 was significantly overexpressed in pancreatic cancer samples, which further demonstrated the cancer-promoting effect of ERAP2 (Figure 5C). In addition, we performed a Kaplan-Meier survival analysis in the TCGA pancreatic cancer dataset, using the median expression of ERAP2 as the threshold. The results are consistent with previous analysis, indicating that high ERAP2 expression is a risk factor for poor prognosis (Figure 5D), while DOCK11 failed to differentiate patient prognosis (Supplementary Figure 1). Finally, we adopted the immunohistochemical data of pancreatic tissues and pancreatic tumors in ProteinAtlas database to verify the expression of the protein level of ERAP2, and further confirmed the high expression of ERAP2 in pancreatic tumor (Figure 5E). The above results suggest that ERAP2 is overexpressed in pancreatic cancer, resulting in a poor prognosis.

### Expression of ERAP2 was positively correlated with gemcitabine resistance

**Figure 6:**
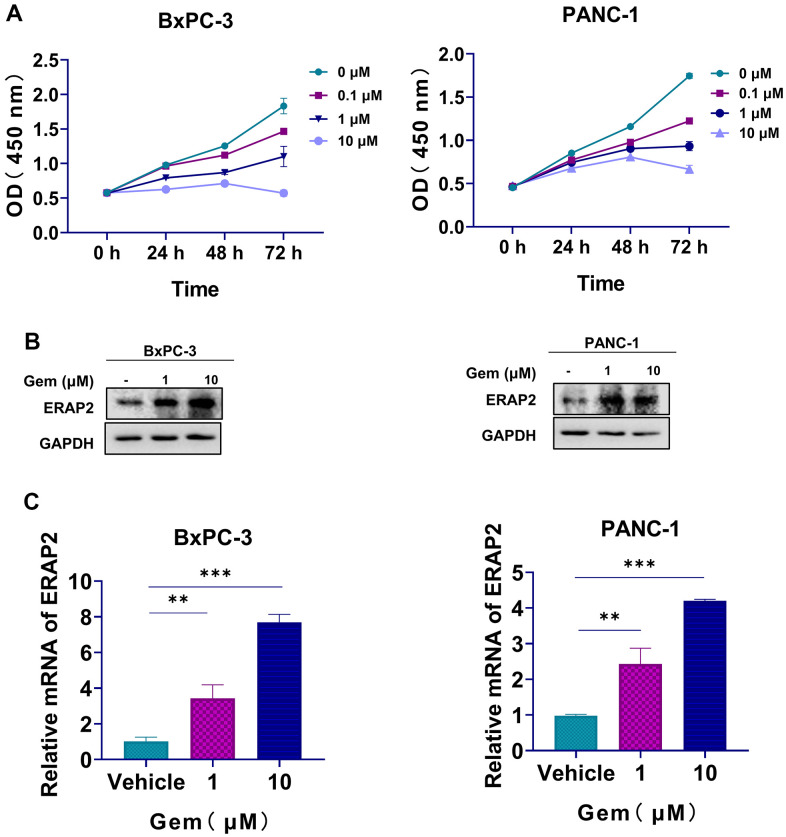
**The relationship between ERAP2 expression and gemcitabine resistance.** (**A**) Cell viability assay in BxPC-3 and PANC-1cell lines treated with gemcitabine. (**B**) The Protein levels of ERAP2 in BxPC-3 and PANC-1 cell lines treated with gemcitabine were assessed by western blot. (**C**) The mRNA levels of ERAP2 in BxPC-3 and PANC-1cell lines treated with gemcitabine were quantified by qRT-PCR. Data were shown as the mean ± SD of three independent experiments (*p < 0.05, **p < 0.01 and ***p < 0.001).

Next, we examined the viability of BxPC-3 and PANC-1 cells exposed to gemcitabine for 0, 24, 48 and 72 h. We discovered that gemcitabine reduced the proliferation of BxPC-3 and PANC-1 cells with IC50 of 10.49 μM and 17.7 μM (72 h) (Figure 6A). We then examined the ERAP2 levels in pancreatic cancer cell lines with gemcitabine treatment. As shown in Figure 6B, 6C, ERAP2 expression and transcription level were significantly increased after gemcitabine treatment. We conclude that ERAP2 expression may play an important role in the sensitivity of pancreatic cancer cells to gemcitabine.

### Inhibition of ERAP2 attenuated the tumorigenesis and increased sensitivity to gemcitabine of pancreatic cancer cells

**Figure 7:**
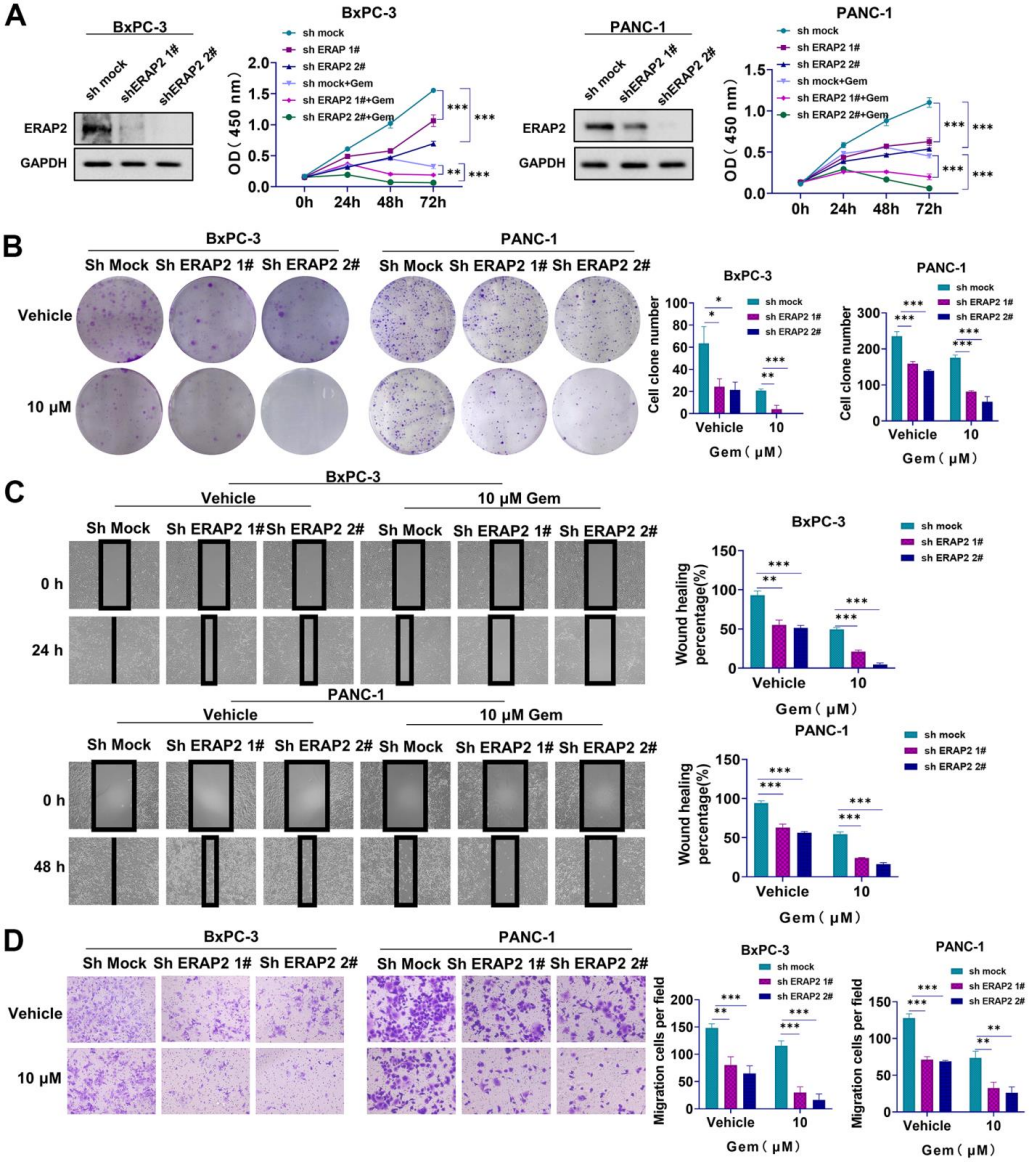
**Effect of ERAP2 knockdown on tumorigenesis and gemcitabine sensitivity in pancreatic cancer cell lines.** (**A**) Cell viability assay in two pancreatic cancer cell lines treated with ERAP2 knockdown, gemcitabine alone or gemcitabine with ERAP2 knockdown. (**B**) Cell proliferation assay in two pancreatic cancer cell lines treated with ERAP2 knockdown, gemcitabine alone or gemcitabine with ERAP2 knockdown. (**C**) Wound healing assay in two pancreatic cancer cell lines treated with ERAP2 knockdown, gemcitabine alone or gemcitabine with ERAP2 knockdown. (**D**) Transwell invasion assay in two pancreatic cancer cell lines treated with ERAP2 knockdown, gemcitabine alone or gemcitabine with ERAP2 knockdown. Data were shown as the mean ± SEM of three independent experiments (*p < 0.05, **p < 0.01 and ***p < 0.001).

We next identified the tumorigenesis and sensitivity of pancreatic cancer cells to gemcitabine after ERAP2 knockdown. It was indicated that ERAP2 knockdown significantly restrained the growth of pancreatic cancer cells (Figure 7A, 7B). Interestingly, ERAP2 knockdown also significantly increased sensitivity to gemcitabine in these two pancreatic cancer cell lines (Figure 7A, 7B). We also tested the effects of ERAP2 on cell migration and invasion by wound healing assay and Transwell invasion assay. As suggested in Figure 7C, 7D, knockdown of ERAP2 remarkably attenuated the migration ability of pancreatic cancer cell lines, and the invasion ability of pancreatic cancer cell lines was also inhibited. Compared with gemcitabine alone, knockdown ERAP2 combined with gemcitabine further reduced the migration ability of pancreatic cancer cells, and the invasion ability was more significantly inhibited (Figure 7C, 7D). Overall, our results suggest that down-regulation of ERAP2 significantly blocks the tumorigenic ability of pancreatic cancer cells and significantly enhances the anti-pancreatic cancer activity of gemcitabine.

## DISCUSSION

Gemcitabine is a first-line drug in the treatment of pancreatic cancer, but its efficacy is limited due to various factors, such as sun exposure, mutations, and overinsurance [3, 21, 22]. Hence, exploring the mechanisms of gemcitabine resistance is critical to enhance the prognosis of patients with this cancer. We firstly screened out 359 positively and 456 negatively genes that were significantly associated with IC50 values from drug sensitivity data in GDSC, and then performed functional annotation analysis on them. At the same time, we selected 68 patients with pancreatic cancer who received gemcitabine in the TCGA database and performed Cox survival analysis on originally selected drug-sensitive genes. We discovered that ERAP2 and DOCK11 were both related to gemcitabine resistance. Interestingly, ERAP2 promoted gemcitabine resistance, and high ERAP2 expression is a risk factor for poor prognosis. Unfortunately, DOCK11 failed to differentiate patient prognosis.

ERAP2 has previously been considered a homologue of placental leucine aminopeptidase/insulin-regulated aminopeptidase [23–25], but its function has not been thoroughly studied. Recent studies report that ERAP2 is an oncogenic gene, overexpressed in variant cancers, such as glioblastoma, choriocarcinoma and oral cavity squamous cell carcinoma (OSCC) [26–29]. ERAP2 overexpression has been reported to benefit cervical metastasis, resulting in a poor prognosis of OSCC [30]. Meanwhile, ERAP2 promotes tumor immune escape by helping to generate peptide ligands for MHC presentation [31, 32]. Given the importance of ERAP2 in immune evasion of cancer, potential clinical applications of its inhibitors have great prospects in tumor immunotherapy [33]. Inhibition of ERAP2 activity may be an effective method to improve tumor antigenicity, but the specific mechanisms need to be further elucidated. Similarly, we found that ERAP2 also closely relevant to the survival of pancreatic cancer patients. To our knowledge, the area of ERAP2 in gemcitabine drug resistance has never been set foot in. We found that gemcitabine treatment promoted ERAP2 expression, indicating that ERAP2 was involved in gemcitabine resistance. Furthermore, we demonstrated that knockdown of ERAP2 significantly improved the killing effect of gemcitabine on pancreatic cancer cells. Our data showed that ERAP2 played a role the classic PI3K/AKT/mTOR pathway of drug resistance through KEGG pathway analysis (Supplementary Figure 2A and Supplementary Table 1), which was further confirmed by the GEPIA analysis tool (Supplementary Figure 2B, 2C). Additionally, ERAP2 showed a significant down-regulation when mTOR was suppressed in the Connectivity Map tool in the IPA software (Supplementary Figure 2D).

The PI3K/AKT/mTOR signaling pathway is critical in cell physiology and regulates cell growth, survival, metastasis and metabolism by responding to many extracellular stimuli [34–37]. mTOR was reported to mediate drug resistance when activated in diverse tumor. For instance, mTOR activation was reported to promote lapatinib resistance in breast cancers [38]; mTOR modulates gemcitabine resistance in lung cancer through mTORC2 [39]. In pancreatic cancer, activating mTOR can promote glycolysis and reduce gemcitabine sensitivity [40]. At present, mTOR inhibitors such as rapamycin and temsirolimus have been approved for clinical use, and novel mTOR inhibitors are also in clinical research. As a downstream molecule of mTOR, ERAP2 inhibition may have better potential for pancreatic cancer treatment and blockade of gemcitabine resistance. The development of ERAP2 inhibitors will further expand the therapeutic options for pancreatic cancer, and provide strategies for gemcitabine combination therapy.

In summary, we found that ERAP2, a previously unreported biomarker, predicts sensitivity to gemcitabine in patients with pancreatic cancer, possibly acting in part through the PI3K/AKT/mTOR signaling pathway. In clinical treatment, it is reasonable to consider that gemcitabine combined with ERAP2 inhibitor may improve the sensitivity of pancreatic cancer patients to gemcitabine.

## MATERIALS AND METHODS

### Identification of genes related to gemcitabine sensitivity

Drug sensitivity data and transcript data were obtained from the GDSC database. The drug response data includes two datasets, GDSC1 and GDSC2. GSDC1 was a discovery set to obtain genes related to gemcitabine sensitivity, and the final results were validated in GDSC2. The IC50 values of pancreatic cancer cells and their corresponding transcripts were analyzed, and finally 359 genes positively as well as 456 genes negatively correlated with IC50 were obtained.

### Construction of drug-sensitive prognosis model

To further screen out key genes, PAAD transcript and clinical data were collected from the TCGA database for subsequent analysis through the UCSC Xena browser (http://xena.ucsc.edu), and we totally obtained 68 pancreatic cancer patients who received gemcitabine. Finally, 815 candidate genes were analyzed by univariate Cox analysis (359 + 456), and the top 20 genes were taken into the Lasso algorithm to create the final multivariate Cox model. Additionally, we included other 113 pancreatic cancer patients as validation set. The R package glmnet (version 2.0.18) and ggrisk (version 1.2) were used for visualization of these results. The cut off value of risk score was obtained by Xtile software (version 3.6.1).

### Functional annotation and pathway analysis

To investigate a comprehensive set of functionally annotated hub genes, David online analysis tool was utilized to carry out GO function annotation and KEGG pathway analysis on 359 positive correlation genes and 456 negative correlation genes, respectively (https://david.ncifcrf.gov). The regulatory network of ERAP2 is constructed using IPA software.

### The analysis of expression and prognosis

The GEPIA2 online tool (http://gepia2.cancer-pku.cn) was used to analyze the expression and prognosis of the target gene, and the protein level of the gene was further verified in The Human protein atlas database (https://www.proteinatlas.org).

### Cell culture

Pancreatic cancer cell lines PANC-1 and BxPC-3 were grown in Dulbecco's modified Eagle (DMEM, Gibco) with the addition of 10% fetal bovine serum (FBS; ExCell Bio).

### Reagents

Gemcitabine was purchased from Selleck. Anti-GAPDH (1:5000) and ERAP2 (1:1000) antibodies were purchased from Proteintech.

### shRNA transfection

6-well plates were inoculated with PANC-1 and BxPC-3 cells. Add 1 mL of lentiviral supernatant containing sh-ERAP2 (Genechem) lentiviral construct or control vector and 1 mL DMEM media with10% FBS to cells when the cell concentration reaches 50%-60%. Two days after infection, 2 μg/mL puromycin was utilized to screen cells that had been successfully infected. Two days later, the inhibition of ERAP2 was investigated by western blot.

### Cell viability assay

The cells were evenly plated in a 96-well culture plate with 5000 cells per well. The cells were treated with 0, 0.1, 1 and 10 μM gemcitabine on the second day. The cytotoxicity of gemcitabine was determined at 0, 24, 48 and 72 h, respectively. 10 μL CCK-8 reagent (Biotool) was supplemented to each well, and the absorbance was determined at 450 nm after incubation at 37° C for 2 h.

### Quantitative real-time PCR

Total RNA was extracted with MgZol reagent (Magen) according to the instructions. Quantitative real-time PCR was conduct to detect the mRNA level of ERAP2. Primers were designed and synthesized by Sangon Biotech. The primer sequences:

ERAP2:

Forward 5’- GAGGCGGAGTCTTGCTCTGTTG-3’

Reverse 5’- GAGGCAGGAGAATGGCGTGAAC-3’

### Western blot

Cells were washed twice with PBS and collected. The mixture of RIPA buffer (Beyotime) and protease inhibitor (Bimake) was added. The cells were lysed for 30 min and the supernatant was collected by centrifugation. Protein concentration was determined by BCA protein assay kit (Beyotime). Protein was isolated with 10% SDS-PAGE and transferred to PVDF membrane after sufficient protein separation. The PVDF membrane was sealed with 5% skim milk at room temperature for 1 h, cleaned with PBS for 15min, and incubated overnight with primary antibody at 4° C. Subsequently, the PVDF membrane was incubated with secondary antibody at room temperature for 1 h. After cleaning with PBS for 30 min, ECL reagent was used for chemiluminescence detection.

### Colony formation assay

Cells were seeded in 6-well culture plates with 1,000 cells per well. On the second day, cells were treated with 0, 0.1, 1, and 10 μM gemcitabine, respectively. After 24 h, the fresh medium was replaced. The fresh medium was replaced every 3 days thereafter. The cell status was observed after 10-14 days, and the medium was discarded after the cells formed suitable clones. After washed with PBS 2 times, the cells were fixed by 500 μL paraformaldehyde. After 15 min, paraformaldehyde was discarded. After washed with PBS 2 times, the cells were stained by 500 μL crystal violet. After 10 min, crystal violet was discarded. After washed with PBS 2 times, colonies were counted after the six-well plate becomes dry.

### Wound healing assay

Cells were plated in 6-well plates until the density reached 80%-100%. The cells were scratched using a sterile 200μL spear tip. Wash the cells gently with PBS three times without breaking the drawn lines. Then 10 μM gemcitabine was added and cell microscope images were taken at 0, 24 and 48 h. Three different areas were selected from each well and the ability of cells to migrate was assessed according to the degree of healing.

### Cell invasion assay

Cells invasion ability were evaluated using 24-well chemotaxis chambers (Costar, #3422). After pretreatment at 37° C for 12 h with a mixture of 60 ul serum-free medium (7): Matrigel (1) in the upper chamber, cells (50000 cells/well) suspended on 100 μL serum-free medium were added, followed by 500 μL culture base containing 30% FBS in the lower chamber. 24 h later, after washed with PBS 2 times, the cells were fixed by 500 μL paraformaldehyde. After 15 min, paraformaldehyde was discarded. After washed with PBS 2 times, the cells were stained by 500 μL crystal violet. After 10 min, crystal violet was discarded. After washed with PBS 2 times, photographed and counted.

### Data analysis

Data were statistically analyzed by the unpaired two-tailed Student’s t-test of Graphpad Prism software (version 8.0), and *P < 0.05* was considered as statistically significant difference between groups.

## Supplementary Material

Supplementary Figures

Supplementary Table 1
