# Supplementary material for: ERAP2 as a potential biomarker for predicting gemcitabine response in patients with pancreatic cancer
Source: Aging (Albany NY). 2022 Oct 8;14(19):7941–58. doi: 10.18632/aging.204324 (PMC9596206; doi:10.18632/aging.204324)
Supplement: Supplementary Figures [file aging-14-204324-s001.pdf]

SUPPLEMENTARY FIGURES

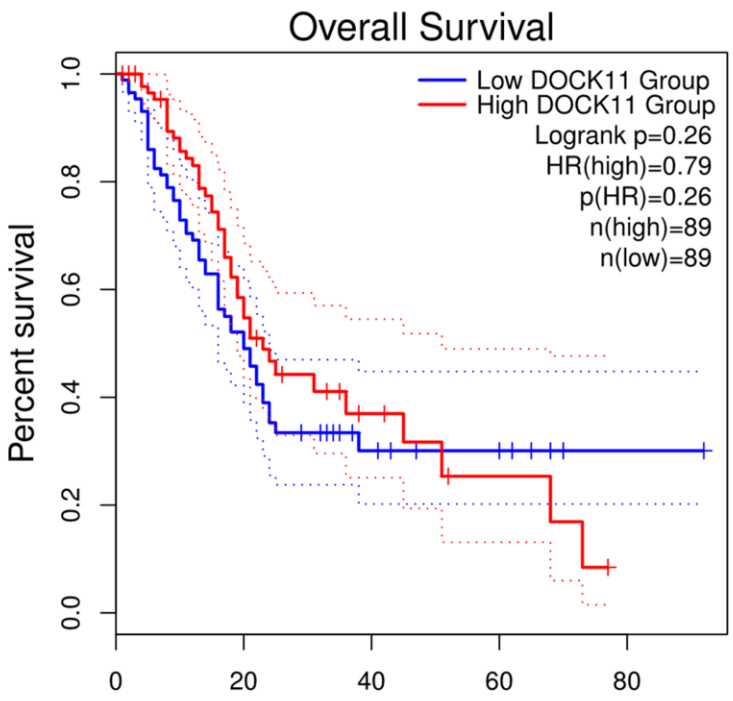

Supplementary Figure 1. Prognosis of DOCK11 in pancreatic cancer. Kaplan-Meier survival curve of DOCK11 in pancreatic cancer data set from GEPIA database.

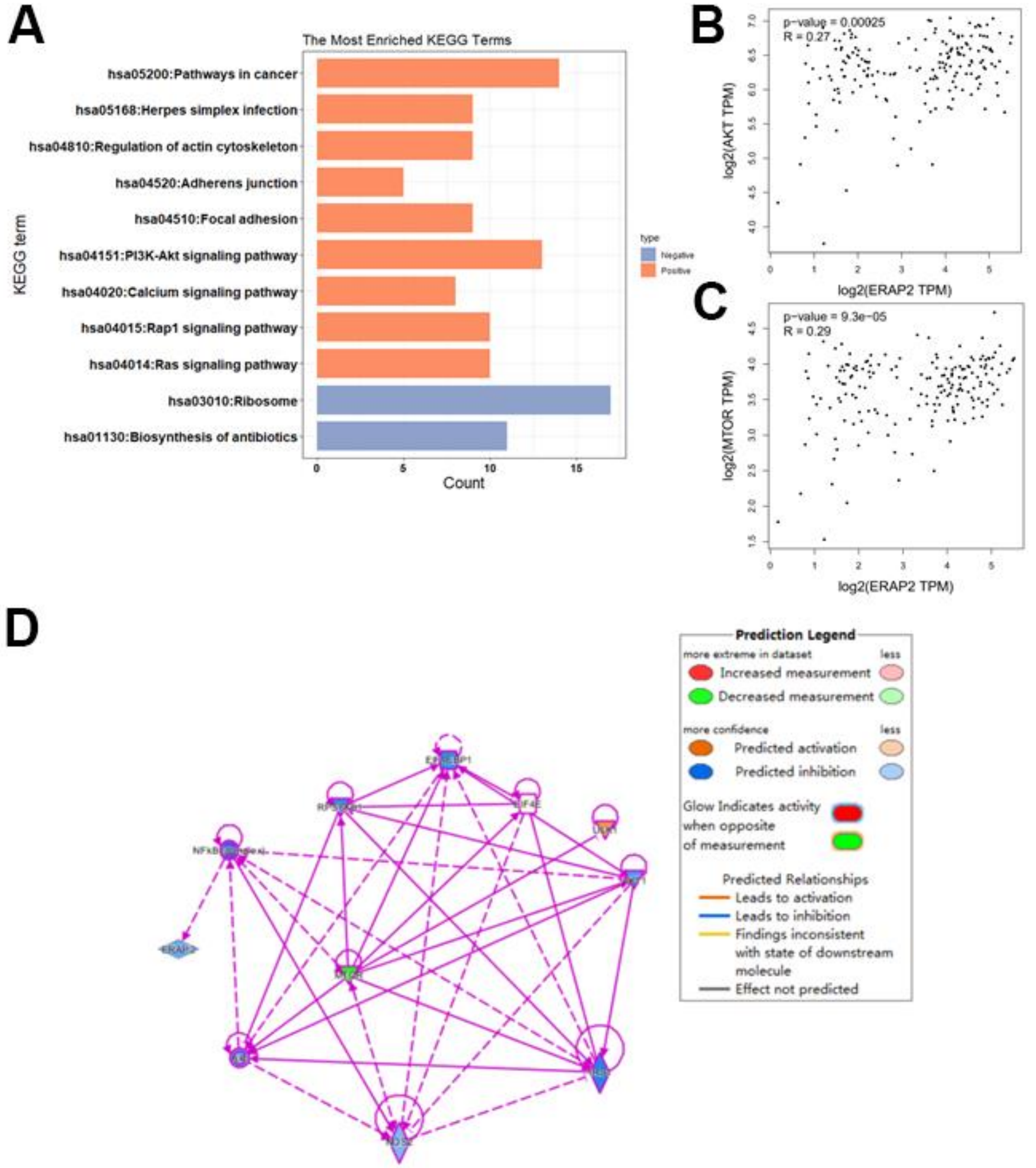

Supplementary Figure 2. ERAP2 involved in the classic AKT/mTOR pathway. (**A**) KEGG pathway analysis to validate the effects of ERAP2. (**B**) The correlation between ERAP2 and AKT was analyzed by GEPIA analysis tool. (**C**) The correlation between ERAP2 and mTOR was analyzed by GEPIA analysis tool. (**D**) The regulatory network of ERAP2 is constructed using IPA software.
