# Supplementary material for: ERAP2 as a potential biomarker for predicting gemcitabine response in patients with pancreatic cancer
Source: Aging (Albany NY). 2022 Oct 8;14(19):7941–58. doi: 10.18632/aging.204324 (PMC9596206; doi:10.18632/aging.204324)
Supplement: Supplementary Table 1 [file aging-14-204324-s002.pdf]

SUPPLEMENTARY TABLE

Supplementary Table 1. Enrichment pathway of gemcitabine sensitivity related-genes.

Term	P value
hsa04015: Rap1 signaling pathway	0.01535
hsa05168: Herpes simplex infection	0.019619
hsa04014: Ras signaling pathway	0.023563
hsa04151: PI3K-Akt signaling pathway	0.025084
hsa05200: Pathways in cancer	0.028822
hsa04510: Focal adhesion	0.036351
hsa04810: Regulation of actin cytoskeleton	0.040021
hsa04520: Adherens junction	0.041712
hsa04020: Calcium signaling pathway	0.047292
hsa03010: Ribosome	1.08E-08
hsa01130: Biosynthesis of antibiotics	0.010473
